# Meningiomas Can Present as Intracranial Hemorrhage in Rare Cases: A Case Report of a Patient With a History of Minor Trauma and Massive Intracranial Hemorrhage

**DOI:** 10.7759/cureus.25823

**Published:** 2022-06-10

**Authors:** Matthew Jenson, Quoc-Han Nguyen, Peter Fiester, Jeet Patel, Dinesh Rao

**Affiliations:** 1 Neuroradiology, University of Florida Health, Jacksonville, USA; 2 Radiology, University of Florida Health, Jacksonville, USA

**Keywords:** ground level fall, adult brain tumor, intracranial hemorrhage, hemorrhage, meningioma

## Abstract

Meningiomas are relatively common intracranial tumors. While typically discovered incidentally or related to symptoms from regional mass effect, on rare occasions, they can present as acute intracranial hemorrhage. We report a case of a 62-year-old male who presented with significant acute intracranial hemorrhage with a history of minor trauma. Imaging workup demonstrated a hemorrhagic mass to be the likely cause of the hemorrhage. Upon resection of the mass, pathology demonstrated meningioma. It is important to thoroughly investigate intracranial hemorrhage, particularly when it appears out of proportion to any known causative event, in order to accurately diagnose, manage, and treat these patients.

## Introduction

Only 3.9% of brain tumors demonstrate spontaneous macroscopic intracranial hemorrhage [1]. Intracranial tumors with spontaneous intracranial hemorrhage are typically associated with metastatic disease from melanoma, bronchogenic carcinoma, choriocarcinoma, renal adenocarcinoma, or oligodendrogliomas and glioblastomas [2].

Meningioma is the most common brain tumor and reportedly accounts for 37.6% of all brain tumors, with an average annual age-adjusted incidence of 8.6 per 100,000 people, and a median age of onset of 66 years [3]. Only 2% of meningiomas have been shown to be associated with spontaneous intracranial hemorrhage [4]. However, even though the rate of spontaneous hemorrhage with meningiomas is low, due to the high incidence, hemorrhagic meningiomas should be considered a potential etiology when patients with intracranial hemorrhage have an atypical presentation or hemorrhage out of proportion to their clinical history.

## Case presentation

**Figure 1:**
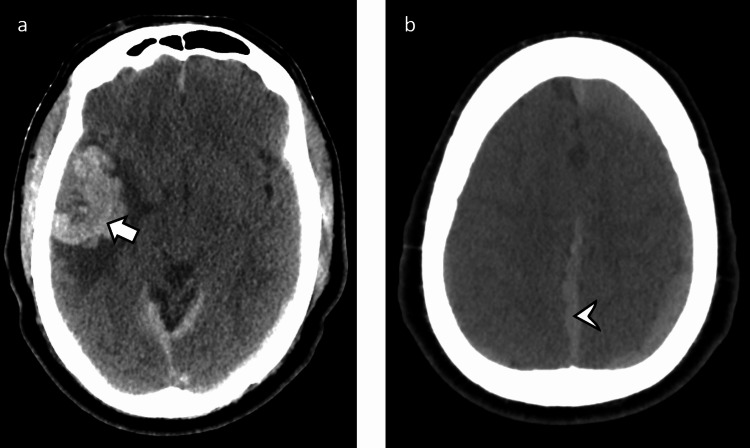
CT head without contrast a: Large right temporal intraparenchymal hemorrhage (arrow) with surrounding edema as well as partially visualized subdural hemorrhages along the anterior falx and bilateral tentorium. b: Subdural hemorrhage along the falx (arrowhead) and bilateral cerebral convexities CT: computed tomography

Our patient was a 62-year-old male who presented to an outside medical facility with a history of a ground-level fall and altered mental status. Initial non-contrast CT head demonstrated what appeared to be a large focus of hemorrhage in the right temporal lobe, surrounding vasogenic edema, and with subdural hematomas along the bilateral cerebral convexities, falx, and tentorium (Figure 1).

**Figure 2:**
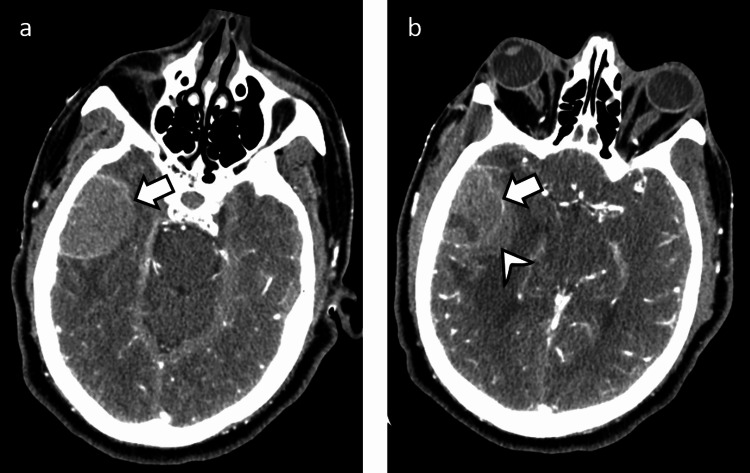
CTA head a: Portions of the right temporal findings appeared very well circumscribed on the CTA head with a possible thin enhancing rim (arrow). b: In other portions, you can see where hemorrhage (arrowhead) has extended beyond the faintly enhancing rim (arrow) CTA: computed tomography angiography

A CTA head was then obtained. While there was no obvious vascular malformation, portions of the right temporal hemorrhage appeared to have a well-circumscribed enhancing rim (Figure 2).

**Figure 3:**
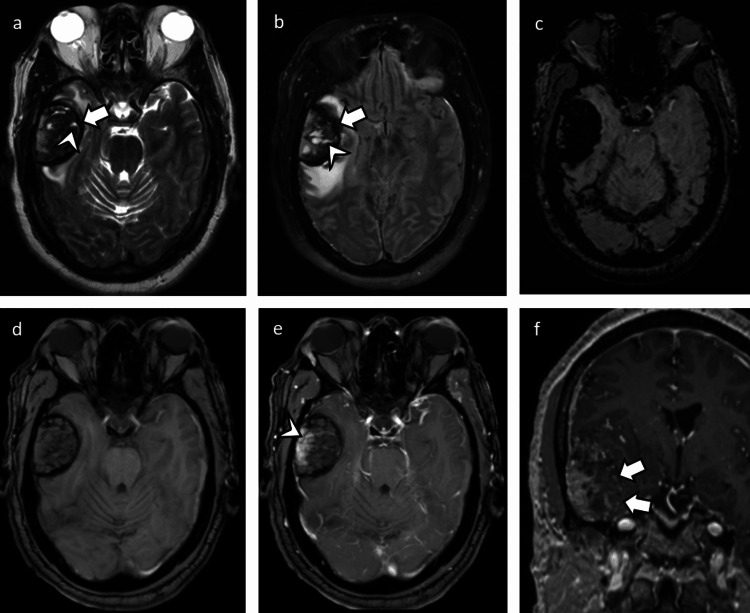
MRI brain without and with contrast a and b: T2 and FLAIR sequences demonstrating a lesion with a T2 hypointense rim (arrows) with surrounding edema. Internal fluid-fluid levels likely related to the internal hemorrhagic component. Additional regions of hemorrhage with fluid-fluid levels (arrowheads) are seen outside of the T2 hypointense rim. c: SWI sequence demonstrating marked susceptibility artifact representative of the hemorrhage, which appears to coincide with the regions that exhibit fluid-fluid levels. d and e: T1 precontrast and postcontrast imaging demonstrate an enhancing component of tumor (arrowhead) along the lateral margin. f: Zoomed-in coronal T1 postcontrast demonstrating enhancing component of the tumor along the lateral margin with the additional suggestion of thin peripheral enhancement (arrows) MRI: magnetic resonance imaging; FLAIR: fluid-attenuated inversion recovery; SWI: susceptibility-weighted imaging

Subsequently, an MRI brain without and with contrast was performed, which demonstrated a 4.6-cm heterogeneous right temporal extra-axial mass (Figure 3) with a T2 hypointense peripheral capsule. This exhibited heterogeneous enhancement with prominent fluid-fluid levels internally, likely due to internal blood products. There did not appear to be a prominent dural tail present, although multiple areas suggested a cleft between adjacent brain tissue and the mass. The subdural blood products along the convexities, falx, and tentorium were again seen as well as additional blood products adjacent to the mass that were likely external to the mass.

The patient underwent a craniotomy for resection of the mass. Pathology demonstrated meningioma, World Health Organization (WHO) grade 1. Postoperative MRI demonstrated postsurgical changes without evidence of residual tumor. The patient’s postoperative course was uneventful and he was eventually discharged home for future follow-up and surveillance.

## Discussion

WHO recognizes 15 histologic subtypes of meningiomas according to cell type. WHO also categorizes meningiomas as either grade I benign, grade II atypical, or grade III malignant based on the rate of growth and likelihood of recurrence. Meningiomas are most commonly slow-growing and progress clinically over a period of months to years [5]. However, meningiomas can be a cause of spontaneous intracranial hemorrhage in rare cases and can present acutely with associated symptoms. Niiro et al. reviewed 298 meningiomas treated over a 10-year period and found that 2% of these presented with a hemorrhagic onset [4].

Hemorrhages associated with meningiomas can be significant with high morbidity and mortality rates. After a review of 143 cases with meningioma-associated spontaneous intracranial hemorrhage, Bosnjak et al. demonstrated an overall mortality rate of 21.1% and a major morbidity rate of 36% [6]. Hemorrhage related to meningiomas has also been shown to occur more likely in patients aged less than 30 years and more than 70 years [6].

In patients with macroscopic hemorrhage related to a meningioma, subarachnoid hemorrhage is the most common type, followed by intraparenchymal and subdural hemorrhages [7]. The percentage of meningiomas that demonstrate microscopic intratumoral hemorrhage is much higher with a rate of 7.9% based on histopathologic analysis [8]. Multiple histologic subtypes have been shown to hemorrhage, though the meningothelial subtype tends to predominate these cases [9].

Proposed mechanisms for macroscopic hemorrhage associated with meningiomas include rupture from abnormal blood vessels, direct vascular invasion by the tumor, tumor infarction, rupture of subdural veins, vasoactive substances released by the tumor, and fragility of vessel walls due to rapid tumor growth [4,10]. However, Lefranc et al. demonstrated that in patients with subdural hemorrhage related to a meningioma, the frequent association of intratumoral hemorrhage makes intratumoral hemorrhage that then secondarily diffuses into the subdural space a probable common culprit [11].

It is important to include the possibility of a hemorrhagic mass in the differential diagnosis to ensure that patients receive appropriate workup and treatment. Sometimes, significant hemorrhagic products can mask the meningioma on initial imaging and can lead to a delay in diagnosis, even after multiple surgeries for hematoma evacuation [11].

## Conclusions

Meningiomas are a rare cause of spontaneous intracranial hemorrhage. Although the tumor may be difficult to appreciate on initial CT imaging, patients with meningioma-related intracranial hemorrhage exhibit high morbidity and mortality. Thus, it is important to include this possibility in the differential diagnosis and pursue MRI imaging so that these patients can receive appropriate management and treatment.
